# Effectiveness and safety of motion style acupuncture treatment for acute neck pain: a multicenter randomized controlled trial

**DOI:** 10.1186/s13020-026-01332-0

**Published:** 2026-01-23

**Authors:** Yoon Jae Lee, Doori Kim, Kyoung Sun Park, Suna Kim, Ji-Yeon Seo, Hyun Woo Cho, In Heo, Woo-Chul Shin, Jae-Heung Cho, Jung-Hyun Kim, Byung-Kwan Seo, In-Hyuk Ha

**Affiliations:** 1 Jaseng Spine and Joint Research Institute, Jaseng Medical Foundation, 540, Gangnam-gu, Seoul, 06110 Republic of Korea; 2 Jaseng Hospital of Korean Medicine, Seoul, Republic of Korea; 3 Daejeon Jaseng Hospital of Korean Medicine, Daejeon, Republic of Korea; 4 Bucheon Jaseng Hospital of Korean Medicine, Bucheon, Republic of Korea; 5 Haeundae Jaseng Hospital of Korean Medicine, Busan, Republic of Korea; 6 School of Korean Medicine, Pusan National University, Yangsan, Republic of Korea; 7 College of Korean Medicine, Kyung Hee University Korean Medicine Hospital, Kyung Hee University, Seoul, Republic of Korea; 8 Department of Acupuncture & Moxibustion, College of Korean Medicine, Kyung Hee University Hospital at Gangdong, Kyung Hee University, Seoul, Republic of Korea

**Keywords:** Acute neck pain, Health-related quality of life, Motion-style acupuncture treatment, Musculoskeletal disorder, Pain relief

## Abstract

**Background:**

Acute neck pain is common and often resolves spontaneously; however, a substantial proportion of cases progress to chronicity, leading to long-term disability and socioeconomic burden. Effective interventions that facilitate rapid recovery during the acute phase remain limited. This study compared the effectiveness and safety of motion-style acupuncture treatment (MSAT) with conventional acupuncture in patients with acute neck pain.

**Methods:**

This multicenter, randomized controlled trial was conducted across four Korean medicine hospitals. A total of 128 adults aged 19–70 years with acute neck pain (≤ 4 weeks) and visual analog scale (VAS) score ≥ 5 at rest or during movement were randomized in a 1:1 ratio to receive MSAT or conventional acupuncture 2–3 times weekly for 2 weeks. Randomization was performed using site-stratified permuted block sequences with variable block sizes, and outcome assessors were blinded to group allocation. The primary outcome was the change in neck pain during movement, measured by the visual analog scale (VAS), from baseline to Week 3. Analyses were conducted according to the intention-to-treat principle.

**Results:**

MSAT resulted in significantly greater reductions in movement-related pain at Week 3 compared with acupuncture (between-group difference: 15.24 mm; 95% CI 9.43–21.05), with effects sustained through Week 9. MSAT also led to greater improvements in neck disability (Neck Disability Index difference at Week 3: 7.49; 95% CI 4.23–10.75) and health-related quality of life (EQ-5D-5L difference at Week 9: − 0.03; 95% CI − 0.06 to 0.00). Seven participants (5.5%; MSAT, n = 3; acupuncture, n = 4) were lost to follow-up or discontinued the intervention. Adverse events were mild and occurred at comparable rates between groups.

**Conclusions:**

MSAT, which integrates acupuncture with guided movement, was associated with more rapid pain relief and functional improvement than conventional acupuncture, without compromising safety. These fundings suggest that MSAT may be a clinically useful option to support early functional recovery in patients with acute neck pain.

*Trial Registration* ClinicalTrials.gov (Identifier: NCT04539184).

**Supplementary Information:**

The online version contains supplementary material available at 10.1186/s13020-026-01332-0.

## Background

Neck pain is a common musculoskeletal disorder with a high annual incidence, reported to range from approximately 10.4% to 21.3% in population-base studies [1]. Incidence rates are substantially higher among occupational groups such as office workers and healthcare professionals, in whom sustained postures and repetitive tasks are common [2]. Consequently, neck pain frequently affects working-age individuals and contributes to work absenteeism, reduced productivity, and increased healthcare utilization. Reflecting this substantial burden, neck pain ranks as the third leading non-fatal condition in Europe in terms of disability-adjusted life years [3].

Although neck pain often initially presents as an acute episode, a considerable proportion of individuals experience persistent or recurrent symptoms. Acute neck pain is typically characterized by sudden onset, movement-provoked pain, and functional limitation, and inadequate resolution during the early phase may increase the risk of progression to chronic neck pain [4, 5]. These observations underscore the importance of effective early-phase interventions aimed at pain relief, functional recovery, and potential mitigation of chronicity.

Current treatment recommendations for acute neck pain generally include pharmacological therapy, exercise, and physical modalities [6]. However, pharmacological options provide limited benefit [7] and may be associated with adverse events [8]. As a result, there is growing interest in non-pharmacological and integrative approaches for managing acute neck pain.

Acupuncture is widely used for neck pain and is recommended in several clinical practice guidelines [9]. While the evidence supporting acupuncture is more robust for chronic neck pain, emerging studies suggest that acupuncture may also improve pain and function in acute musculoskeletal conditions. Nevertheless, high-quality randomized controlled trials focusing specifically on acute neck pain remain limited, and the optimal acupuncture-based strategy during the acute phase has not been clearly established.

Motion-style acupuncture treatment (MSAT) integrates guided active or passive movement into acupuncture during needle retention at symptomatic sites. By combining acupuncture with controlled movement, MSAT aims to directly address movement-related pain and restricted mobility—key clinical features of acute neck pain. Previous clinical studies have reported favorable effects of MSAT in acute musculoskeletal conditions [10], such as acute low back pain [11] and whiplash-associated disorders [12]. However, no randomized controlled trials have directly compared MSAT with conventional acupuncture alone in individuals with acute neck pain.

Therefore, the objective of this study was to evaluate, in a real-world clinical setting, whether MSAT, which integrates guided cervical movement into conventional acupuncture, provides additional and integrative benefits in pain relief, functional recovery, and health-related quality of life compared with acupuncture alone in patients with acute neck pain.

## Methods

### Study design

We recruited 128 participants with acute neck pain and randomly assigned them in a 1:1 ratio to receive either acupuncture or MSAT, with 64 participants per group. The trial was conducted across four Korean medicine hospitals specializing in spinal care. The study protocol was registered with ClinicalTrials.gov (Identifier: NCT04539184), and additional methodological details are provided in the published protocol [13]. The schedule of study visits is shown in Supplementary Table 1. Ethical approval was obtained from the institutional review boards of all participating sites (approval numbers: JASENG 2020–07–014, JASENG 2020–07–015, JASENG 2020–07–016, and JASENG 2020–07–017).

### Participants

The eligibility criteria were: adults aged 19–70 years who had acute neck pain, defined as pain lasting at least 24 h but less than 4 weeks, with either new onset or acute exacerbation of existing neck pain, a visual analog scale (VAS) score ≥ 5 at rest or during movement, and who provided written informed consent. Acute neck pain was clinically classified as neck pain with mobility deficits, characterized by localized neck pain and movement-related pain or restriction without headache or radiating arm pain, with symptom duration of less than 4 weeks, according to the 2017 Clinical Practice Guidelines [14]. The exclusion criteria included pain-related conditions such as spinal metastases, fractures, or dislocations; progressive or severe neurological deficits; soft tissue disorders (e.g., fibromyalgia, rheumatoid arthritis, or gout); chronic illnesses (e.g., cardiovascular disease, kidney disease, dementia, diabetic neuropathy, or epilepsy); current use of steroids, immunosuppressants, or psychotropic medications; contraindications to acupuncture (e.g., bleeding disorders, severe diabetes, or anticoagulant use); prior nonsteroidal anti-inflammatory drug use or acupuncture within the past 3 days; cervical surgery within the past 3 months; recent motor vehicle accident (within 1 month); current pregnancy or plans to conceive; participation in another clinical trial within the past month or anticipated within the next 6 months; inability to provide informed consent; or other clinical concerns at the discretion of the investigator.

### Randomization and blinding

Randomization codes were generated by a statistician using R Studio version 1.1.463 (2009–2018 RStudio, Inc.) with permuted block sizes of 2, 4, and 6 to ensure allocation balance. The randomization sequence was placed into sequentially numbered, opaque, sealed envelopes and delivered to each participating institution, where they were stored in a double-locked cabinet.

Randomization was conducted only after participants provided written informed consent. At the time of allocation, the next sequential envelope was opened in the presence of the participant, and the participant was assigned to the corresponding group. After opening, the envelope was stored separately in a double-locked cabinet. The allocation sequence was concealed until assignment and could not be accessed in advance or modified after allocation.

Because of the nature of the interventions, blinding of participants and treating physicians was not feasible; however, outcome assessors remained blinded throughout the study.

### Intervention

Participants in both the MSAT and acupuncture groups received 2–3 treatments per week for 2 weeks. The total number of sessions was determined by the treating physician based on pain severity. In the acupuncture group, 6–12 disposable sterile needles (30 mm × 0.25 mm; Dong-bang Acupuncture) were inserted at acupoints SI15, TE15, LI16, GB20, BL10, SI14 (bilateral), and GV14 (unilateral), selected according to the physician’s clinical judgment. Needles were inserted either perpendicularly or obliquely depending on the acupoint location, and manual stimulation using a twirling technique was applied to elicit the deqi sensation. Needles were retained for approximately 15 min per session.

In the MSAT group, participants were seated while acupuncture was administered bilaterally at TE15, SI15, and LI16 using disposable sterile needles (30 mm × 0.25 mm; Dong-bang Acupuncture) inserted to a depth of 5–10 mm. The same local cervical–shoulder acupoints (TE15, SI15, and LI16) were used in both the MSAT and acupuncture groups; the key distinction between the two interventions was the incorporation of guided cervical movement during needle retention in the MSAT group. The cervical range of motion (ROM) was assessed; if restriction was observed, the physician facilitated both active and passive neck movements within a pain-free and physiologically safe range by placing one hand on the face of the participant and the other on the posterior neck. Repetitive movements were carefully and physician-controlled performed, followed by reassessment of ROM. If normal ROM was not restored, additional left-to-right and right-to-left guided movements were applied. Throughout the procedure, participants were closely monitored for any adverse events. Each session lasted approximately 15 min.

The control acupuncture group was designed to represent conventional clinical acupuncture practice using commonly applied local acupoints, without guided movement. By matching acupoint selection, treatment frequency, session duration, and physician involvement between groups, the study aimed to evaluate the additional and integrative effects of incorporating guided cervical movement into acupuncture under conditions reflecting routine clinical practice, rather than to distinguish specific from non-specific acupuncture effects.

All treatments, including MSAT and conventional acupuncture, were administered by licensed Korean medicine doctors with more than 3 years of clinical experience who had received standardized training in both Korean medicine treatments and the MSAT protocol.

### Outcomes

The primary outcome was the change in VAS score for neck pain during movement from baseline to Week 3. VAS and numerical rating scale (NRS) scores for pain at rest and during movement were assessed at baseline and at weeks 1, 2, 3, and 9. Pain intensity was assessed using the VAS and NRS, which have demonstrated feasibility, reliability, and associations with multi-item patient-reported outcome measures in neck pain research [15]. The validated Korean version of the Vernon–Mior Neck Disability Index (NDI) [16] and Northwick Park Neck Pain Questionnaire (NPQ) [17], validated in Korean [18], were also assessed at these time points to evaluate neck-related disability. Health-related quality of life was assessed using the Korean version of the EQ-5D-5L [19] and validated Korean version of the 12-Item Short Form Survey (SF-12) [20].

### Sample size

The effect size (Cohen’s d = 0.55) was derived from an unpublished pilot study and was used as the anticipated standardized mean difference between groups for sample size estimation. Assuming a two-sided α of 0.05 and 80% power, the initial sample size was calculated using a two-sample t-test, yielding a total of 106 participants. Because the primary analysis was planned using ANCOVA, the estimate was further adjusted by incorporating the correlation between baseline and the primary endpoint (r = 0.24) observed in the pilot study, resulting in a minimum required sample size of 100 participants. To account for an anticipated dropout rate of 20%, the final target sample size was set at 128 participants. Randomization was stratified by study center to ensure balanced allocation across the four participating institutions. Sample size calculations were performed using G*Power version 3.1.9.7.

### Statistical analysis

The primary analysis was conducted according to the intention-to-treat principle, while the per-protocol analysis included only participants who completed a pre-specified minimum number of treatment sessions. Missing data were addressed using multiple imputation via the Markov Chain Monte Carlo method to generate 20 imputed datasets. In addition, a sensitivity analysis using the last observation carried forward approach was performed.

For continuous outcomes (NRS, VAS, NDI, NPQ, EQ-5D-5L, and SF-12), changes from baseline to each time point were analyzed using ANCOVA, with the baseline value included as a covariate and group assignment as a fixed factor. Linear mixed models were also used to account for repeated measures. To evaluate the overall burden of pain and disability over the study period, area under the curve (AUC) analysis was conducted. To assess the rate of recovery, survival analysis was performed using VAS scores during movement, with recovery defined as a reduction to < 50% of the baseline score. A ≥ 50% reduction in pain intensity has been used as a clinically meaningful responder threshold in previous pain research [21]. Kaplan–Meier curves and log-rank tests were used to compare groups, and hazard ratios were estimated using Cox proportional hazards models. Pre-specified subgroup analyses of the primary outcome were conducted using ANCOVA models with adjustment for the baseline value of the outcome. Treatment-by-subgroup interaction terms were included to assess potential effect modification by baseline characteristics. All statistical analyses were performed using SAS version 9.4 (SAS Institute, Cary, NC, USA), with statistical significance set at P < 0.05.

## Results

**Fig. 1:**
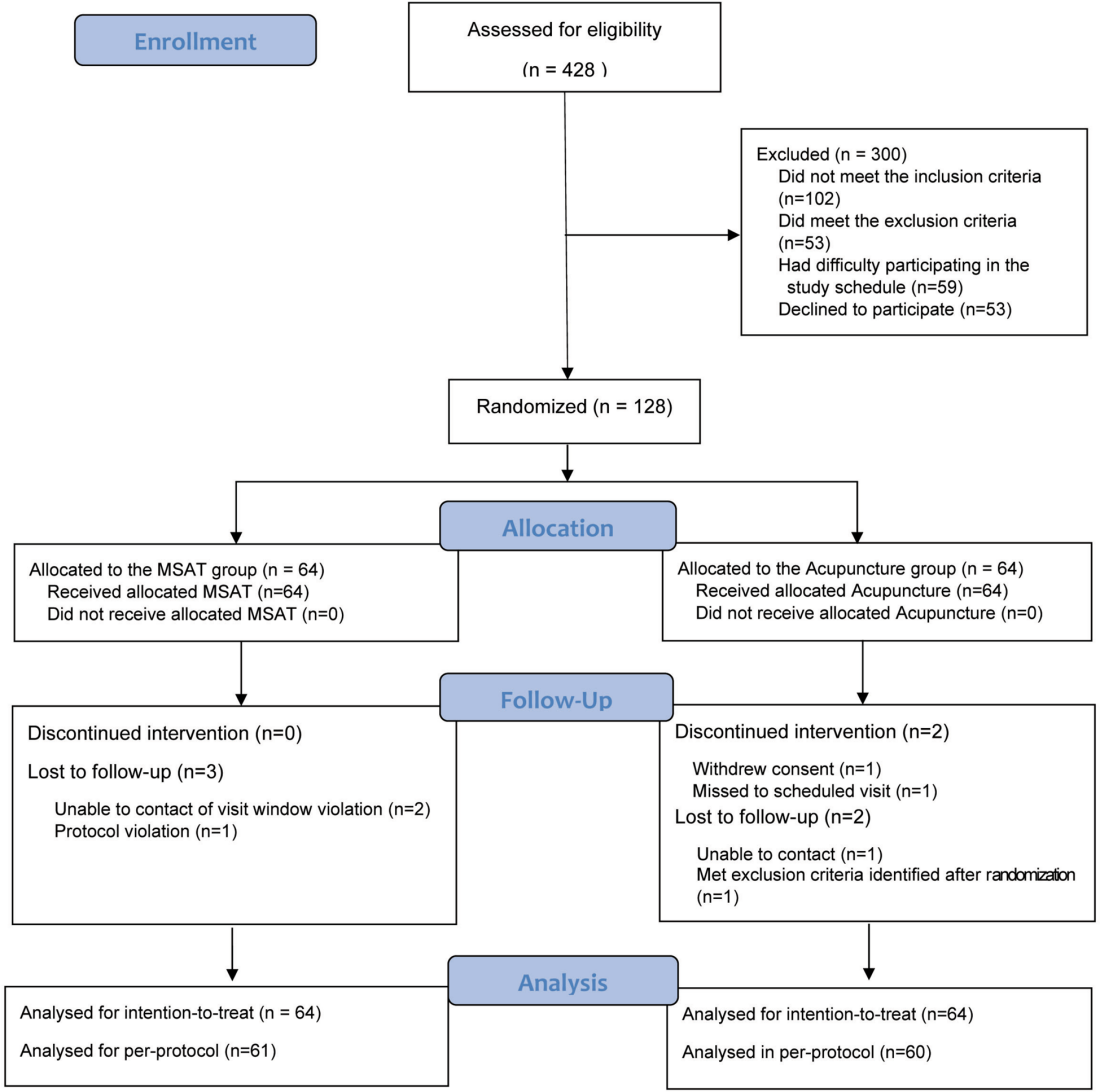
CONSORT flow diagram

Between September 2020 and November 2021, 428 individuals were screened, of whom 300 were excluded owing to not meeting the inclusion or exclusion criteria. The remaining 128 participants were randomized to the MSAT or acupuncture group. In the MSAT group, three participants were lost to follow-up. In the acupuncture group, four participants discontinued or were lost to follow-up, including two who discontinued the intervention and two who were lost to follow-up. Consequently, all 128 participants were included in the intention-to-treat analysis, while 121 participants were included in the per-protocol analysis (Fig. 1).

### Patient characteristics

Baseline demographic and clinical characteristics did not differ significantly between groups (Table 1, Supplement Table 2). Baseline measures of pain, function, and quality of life were also comparable, as was the treatment expectation score, which averaged 6.8 ± 1.3 and 6.8 ± 1.4 in the MSAT and acupuncture groups, respectively.

**Table 1:** Baseline characteristics of participants by randomized group

	MSAT (n = 64)	Acupuncture (n = 64)	P value
Sex			
Female	41 (64.1)	36 (56.2)	0.47
Male	23 (35.9)	28 (43.8)	
Age, mean (SD), year	41.5 (11.8)	39.9 (13.5)	0.518
Height, mean (SD), cm	166.0 (8.6)	165.9 (9.2)	0.934
Body weight, mean (SD), kg	67.1 (16.4)	65.4 (12.4)	0.523
BMI, mean (SD)	24.2 (4.9)	23.7 (3.4)	0.602
X-ray (%)			
Straightening	53 (82.8)	52 (81.2)	1
Narrowing	15 (23.4)	12 (18.8)	0.665
Degeneration	2 (3.1)	6 (9.4)	0.273
Pain duration, mean (SD), days	16.9 (7.3)	16.2 (8.0)	0.602
Medication use for the current episode	4 (6.2)	2 (3.1)	0.68
Previous treatment experience			
MSAT	1 (1.6)	1 (1.6)	1
Acupuncture	49 (76.6)	49 (76.6)	
Both	2 (3.1)	1 (1.6)	
None	12 (18.8)	13 (20.3)	
Treatment preference			
MSAT	19 (29.7)	18 (28.1)	0.119
Acupuncture	5 (7.8)	13 (20.3)	
No preference	40 (62.5)	33 (51.6)	
VAS on movement, mean (SD), mm	65.6 (8.1)	63.4 (12.6)	0.253
VAS at rest, mean (SD), points	52.5 (18.9)	52.1 (14.4)	0.875
NRS on movement, mean (SD), points	6.5 (0.9)	6.3 (1.2)	0.518
NRS at rest, mean (SD), points	5.2 (1.8)	5.1 (1.4)	0.708
NDI, mean (SD), points	32.0 (11.3)	32.6 (10.1)	0.762
NPQ, mean (SD), points	38.9 (12.0)	41.6 (8.8)	0.155
EQ-5D-5L score, mean (SD), points	0.76 (0.11)	0.77 (0.08)	0.519
EQ-VAS, mean (SD), mm	62.3 (14.1)	62.7 (18.9)	0.911
ROM of flexion	40.7 (7.0)	41.4 (7.4)	0.58
ROM of extension	40.5 (7.7)	41.4 (7.2)	0.5
ROM of left rotation	80.2 (13.7)	80.8 (14.1)	0.825
ROM of right rotation	79.9 (13.3)	82.5 (11.4)	0.242
ROM of left lateral flexion	37.8 (8.9)	38.9 (10.3)	0.521
ROM of right lateral flexion	38.1 (8.9)	38.4 (10.1)	0.853
SF-12 score, mean (SD), points			
PCS	44.3 (6.2)	44.8 (7.0)	0.652
MCS	46.5 (10.1)	46.5 (10.5)	0.984

*MSAT* Motion Style Acupuncture Treatment, AT Acupuncture Treatment, *VAS* Visual Analog Scale, *NRS* Numeric Rating Scale, *EQ-5D*-*5L* EuroQol 5-Dimension 5-level, *SF-12* 12-item Short-Form Health Survey, *PCS* Physical Component Summary, *MCS* Mental Component Summary, *EQ-VAS* EuroQol-5 dimension visual analog scale, *NDI*: Neck Disability Index, *NPQ* Northwick Park Neck Pain Questionnaire, *SD* Standard deviation, *BMI* Body mass index, *ROM* Range of Movement

**Table 2:** Primary and secondary outcomes by treatment and time since randomization

Assessment	Categories	Week 1	Week 2	Week 3	Week 9
VAS on movement	MSAT	45.93 (42.84 to 49.03)	28.91 (25.07 to 32.76)	22.99 (18.91 to 27.07)	18.83 (14.38 to 23.27)
	AT	53.68 (50.52 to 56.84)	43.37 (39.48 to 47.27)	38.23 (34.11 to 42.34)	33.06 (28.65 to 37.46)
	Difference	7.74 (3.30 to 12.18)	14.46 (8.97 to 19.95)	15.24 (9.43 to 21.05)	14.23 (7.96 to 20.50)
	*P*-value	< 0.001***	< 0.001***	< 0.001***	< 0.001***
VAS at rest	MSAT	39.43 (36.52 to 42.33)	23.36 (19.90 to 26.82)	18.13 (14.27 to 21.98)	14.32 (9.97 to 18.67)
	AT	43.94 (40.98 to 46.90)	34.27 (30.75 to 37.80)	30.54 (26.66 to 34.43)	26.42 (22.07 to 30.77)
	Difference	4.52 (0.37 to 8.66)	10.91 (5.97 to 15.85)	12.42 (6.93 to 17.90)	12.10 (5.95 to 18.25)
	*P*-value	0.033*	< 0.001***	< 0.001***	< 0.001***
NRS on movement	MSAT	4.63 (4.32 to 4.94)	2.93 (2.56 to 3.30)	2.32 (1.92 to 2.72)	1.85 (1.42 to 2.29)
	AT	5.30 (4.98 to 5.62)	4.25 (3.87 to 4.63)	3.69 (3.28 to 4.09)	3.20 (2.76 to 3.63)
	Difference	0.67 (0.22 to 1.12)	1.32 (0.78 to 1.85)	1.37 (0.80 to 1.94)	1.34 (0.73 to 1.96)
	*P*-value	0.004**	< 0.001***	< 0.001***	< 0.001***
NRS at rest	MSAT	3.95 (3.67 to 4.22)	2.35 (2.00 to 2.69)	1.73 (1.35 to 2.11)	1.35 (0.92 to 1.79)
	AT	4.38 (4.11 to 4.66)	3.44 (3.09 to 3.79)	3.06 (2.67 to 3.44)	2.57 (2.14 to 3.01)
	Difference	0.44 (0.05 to 0.82)	1.10 (0.61 to 1.59)	1.32 (0.78 to 1.87)	1.22 (0.61 to 1.83)
	*P*-value	0.028*	< 0.001***	< 0.001***	< 0.001***
NDI	MSAT	22.85 (20.99 to 24.71)	15.08 (13.08 to 17.09)	14.45 (12.15 to 16.75)	13.55 (10.92 to 16.19)
	AT	24.46 (22.58 to 26.34)	20.49 (18.45 to 22.52)	21.94 (19.62 to 24.25)	18.36 (15.75 to 20.97)
	Difference	1.61 (−1.04 to 4.26)	5.40 (2.54 to 8.26)	7.49 (4.23 to 10.75)	4.81 (1.11 to 8.50)
	*P*-value	0.231	< 0.001***	< 0.001***	0.011*
NPQ	MSAT	−	−	18.87 (16.35 to 21.39)	17.31 (14.12 to 20.51)
	AT	−	−	26.41 (23.90 to 28.93)	23.10 (19.92 to 26.28)
	Difference	−	−	7.54 (3.96 to 11.13)	5.79 (1.28 to 10.29)
	*P*-value	−	−	< 0.001***	0.012*
EQ-5D-5L	MSAT	−	−	0.84 (0.82 to 0.86)	0.85 (0.83 to 0.88)
	AT	−	−	0.82 (0.80 to 0.83)	0.82 (0.80 to 0.84)
	Difference	−	−	−0.02 (−0.05 to 0.01)	−0.03 (−0.06 to 0.00)
	*P*-value	−	−	0.113	0.033*
EQ-VAS	MSAT	−	−	74.82 (70.97 to 78.67)	77.03 (73.41 to 80.65)
	AT	−	−	67.92 (64.04 to 71.79)	75.38 (71.75 to 79.01)
	Difference	−	−	−6.91 (−12.33 to −1.48)	−1.65 (−6.77 to 3.47)
	*P*-value	−	−	0.013*	0.525
PCS (SF-12)	MSAT	−	−	48.87 (47.35 to 50.38)	49.99 (48.40 to 51.57)
	AT	−	−	46.31 (44.80 to 47.82)	48.66 (47.05 to 50.26)
	Difference	−	−	−2.56 (−4.71 to −0.41)	−1.33 (−3.58 to 0.92)
	*P*-value	−	−	0.020*	0.243
MCS (SF-12)	MSAT	−	−	50.67 (48.71 to 52.63)	51.28 (49.46 to 53.11)
	AT	−	−	49.95 (47.98 to 51.93)	51.07 (49.26 to 52.88)
	Difference	−	−	−0.72 (−3.52 to 2.09)	−0.22 (−2.80 to 2.36)
	*P*-value	−	−	0.614	0.868
PGIC	MSAT	−	−	2.01 (1.80 to 2.21)	2.20 (1.96 to 2.44)
	AT	−	−	2.79 (2.59 to 3.00)	2.61 (2.38 to 2.85)
	Difference	−	−	−0.79 (−1.08 to −0.49)	−0.41 (−0.75 to−0.08)
	*P*-value	−	−	< 0.001***	0.016*

*MSAT* Motion Style Acupuncture Treatment, AT Acupuncture Treatment, *VAS* Visual Analog Scale, *NRS* Numeric Rating Scale, *EQ-5D*-*5L* EuroQol 5-Dimension 5-level, *SF-12* 12-item Short-Form Health Survey, *PCS* Physical Component Summary, *MCS* Mental Component Summary, *PGIC* Patient Global Impression of Change, *EQ-VAS* EuroQol-5 dimension visual analog scale, *NDI* Neck Disability Index, *NPQ* Northwick Park Neck Pain Questionnaire

### Treatment

Participants in both groups received an average of 5.5 treatment sessions (MSAT: 5.5 ± 0.8; acupuncture: 5.5 ± 1.0). Detailed information regarding acupoint selection is provided in Supplementary Table 3.

### Primary outcome

**Fig. 2:**
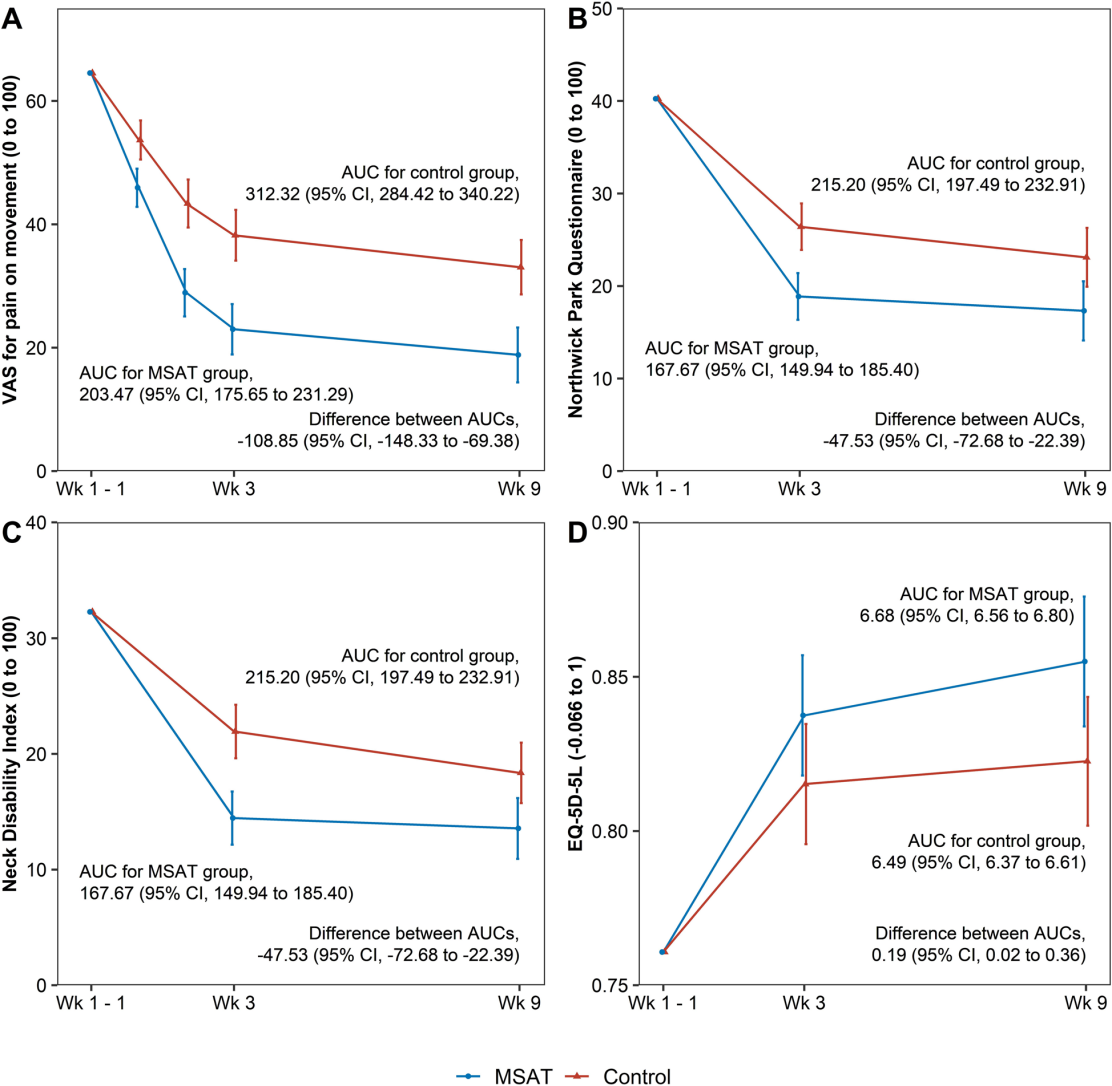
Changes in outcomes over time and area under the curve analyses

At Week 3, the change in VAS score for neck pain during movement showed a significant between-group difference of 15.24 points (95% CI 9.43–21.05), favoring the MSAT group. This significant difference was evident as early as Week 1 and persisted through Week 9 (difference at Week 9:14.23 [95% CI 7.96–20.50]) (Table 2, Fig. 2).

### Secondary outcomes

VAS at rest showed significant between-group differences beginning at Week 1. At Weeks 3 and 9, the differences were 12.42 (95% CI 6.93–17.90) and 12.10 (95% CI 5.95–18.25), respectively, favoring the MSAT group. NRS scores for pain at rest and during movement followed trends consistent with those observed in the VAS outcomes. The NDI also showed significant improvement from Week 2 onward, with between-group differences of 7.49 (95% CI 4.23–10.75) at Week 3 and 4.81 (95% CI 1.11–8.50) at Week 9. Similarly, NPQ scores revealed significant between-group differences at Weeks 3 and 9.

Significant differences were also observed between groups in the EQ-VAS and physical component score (PCS) of the SF-12 at Week 3 and in EQ-5D-5L index scores at Week 9. Patient Global Impression of Change scores differed significantly between groups at Weeks 3 and 9, with mean differences of − 0.79 (95% CI − 1.08 to − 0.49) and − 0.41 (95% CI − 0.75 to − 0.08), respectively (Table 2).

Significant between-group differences in cervical ROM during flexion were observed at Weeks 2 and 3 but not at other time points. Work productivity impairment, assessed using the Work Productivity and Activity Impairment questionnaire, was significantly lower in the MSAT group from Weeks 2 to 9 (Supplementary Table 4).

Sensitivity analyses using the last observation carried forward method showed no substantial deviation from the main results. Consistent trends were confirmed using both linear mixed model and per-protocol analyses (Supplementary Tables 5–7).

AUC analyses comparing cumulative outcomes over time revealed significant between-group differences in pain (VAS, NRS), function (NDI, NPQ), and quality of life measures (EQ-5D-5L, EQ-VAS, and SF-12 PCS) (Supplementary Table 8, Fig. 2).

### Survival analysis

**Fig. 3:**
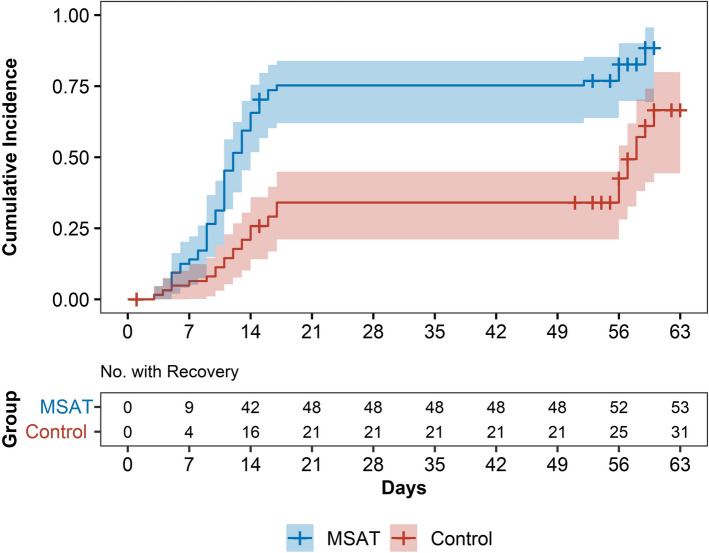
Cumulative incidence of recovery by treatment group

The time to achieve ≥ 50% reduction in VAS during movement was significantly shorter in the MSAT group (median 12 d) than in the acupuncture group (median 58 d; P < 0.001 on the log-rank test). Cox regression analysis yielded a hazard ratio of 2.74 (95% CI 1.74–4.31), indicating significantly faster improvement in the MSAT group (Fig. 3).

### Safety

In the MSAT and acupuncture groups, 15 and 16 adverse events (AEs) were reported, respectively. One AE in the MSAT group and two in the acupuncture group, all involving headache, were considered possibly related to the intervention but resolved spontaneously within 1 day without the need for additional treatment. One serious AE, a case of COVID-19 requiring hospitalization for 11 days, was reported but deemed unrelated to the study intervention.

## Discussion

Although MSAT has been applied to several chronic conditions, most randomized controlled trials to date have focused on its use in acute musculoskeletal disorders. This emphasis likely stems from the frequent presentation of sudden movement limitations and functional impairment in acute conditions, which MSAT is uniquely positioned to address by facilitating rapid mobility restoration. The effectiveness of MSAT has been demonstrated in patients with acute traffic-related injuries [12], and the therapy is currently recommended for treating whiplash-associated disorders in the Korean Clinical Practice Guidelines for Traffic Injury Syndromes [22].

Most randomized controlled trials evaluating MSAT have compared its effectiveness with analgesics [11] or included MSAT only in the intervention arm, while both groups received integrative Korean medicine [12]. Although these studies reported improved functional recovery with MSAT, none have directly compared it with conventional acupuncture in individuals with acute neck pain. Therefore, we evaluated whether MSAT produces distinct clinical effects compared with acupuncture alone in this patient population.

Our findings indicate that MSAT led to significantly greater reductions in pain and improvements in function than acupuncture. These between-group differences emerged as early as Week 1 and persisted through Week 9. Furthermore, survival analysis comparing the time to achieve a 50% reduction in movement-related pain showed a significantly shorter median recovery time in the MSAT group. AUC analyses of pain, functional outcomes, and quality of life across the study period further confirmed the sustained and consistent benefits of MSAT.

Although improvements in cervical ROM were only partially observed, significant between-group differences were identified in functional measures such as the NDI and NPQ. These instruments primarily assess perceived pain intensity and its interference with daily activities rather than isolated joint mobility. This suggests that the observed functional improvements were closely associated with pain relief. Consistent with these findings, improvements in quality of life were also reported. However, the timing of significant changes varied across different quality-of-life instruments. Specifically, the EQ-VAS and PCS of the SF-12 showed significant differences at Week 3, whereas the EQ-5D-5L index score showed significant improvement at Week 9. The early response in EQ-VAS may reflect subjective perception of overall health status among participants, while discrepancies between the EQ-5D-5L and SF-12 PCS likely reflect differences in domain-specific content. The EQ-5D-5L includes functional domains such as mobility, self-care, and usual activities, whereas the SF-12 PCS captures broader constructs such as perceived physical health and stair-climbing ability, which may detect improvements more rapidly.

Unlike conventional acupuncture, MSAT involves needle retention during guided cervical movement, which may raise concerns regarding procedural safety. In particular, potential risks such as needle deformation or breakage could be anticipated when active or passive movement is combined with acupuncture. However, no MSAT-related adverse events, including needle bending or breakage, were observed in the present study. This favorable safety profile may be attributed to several factors. MSAT was performed according to a standardized protocol by trained physicians, with cervical movements carefully controlled within a pain-free range. Continuous physician supervision during the procedure enabled real-time adjustment of needle direction and depth in response to patient movement, thereby minimizing mechanical stress.

Motion style acupuncture treatment (MSAT) can be applied using different procedural approaches. One approach combines acupuncture at the symptomatic region with guided active or passive movement performed during needle retention, whereas another approach applies acupuncture at distal acupoints followed by movement without needle retention at the site of symptoms. In the present study, MSAT was applied using local cervical–shoulder acupoints (TE15, SI15, and LI16), which were also used in the conventional acupuncture group. This selection was based on the clinical characteristics of acute neck pain with mobility deficits, in which pain and functional limitation are predominantly provoked by cervical motion and localized muscle guarding. Applying acupuncture directly to the symptomatic region while introducing guided cervical movement may provide more immediate and targeted sensorimotor input during motion, thereby facilitating early modulation of movement-related pain and restricted mobility. Notably, previous studies reporting favorable effects of MSAT in acute musculoskeletal pain conditions have predominantly applied the intervention at or near the site of pain, supporting the relevance of a localized MSAT approach in acute conditions [11, 12].

The mechanisms underlying the therapeutic effects of MSAT warrant further investigation; however, several plausible pathways may be considered. Acute neck pain is frequently accompanied by fear-avoidance behaviors, in which pain anticipation leads to guarded movement, restricted range of motion, and secondary functional limitation. In this context, MSAT represents an innovative intervention that integrates acupuncture with physician-guided active and passive cervical movement, rather than relying on acupuncture alone.

Acupuncture is thought to modulate pain through central mechanisms, including activation of descending inhibitory pathways, engagement of diffuse noxious inhibitory control, and endogenous opioid release [10]. The incorporation of guided movement during needle retention may complement these effects by directly addressing movement-related fear and facilitating gradual re-exposure to previously avoided motions. Physician guidance and verbal reassurance during movement may help recalibrate pain perception and restore confidence in cervical mobility, thereby interrupting the pain–avoidance cycle.

Importantly, the guided movement component of MSAT may contribute independently to early functional improvement by promoting sensorimotor reactivation and coordination, while its combination with acupuncture may produce synergistic effects on pain modulation. Rather than acting primarily through structural tissue changes, the rapid improvements observed in this study are more plausibly explained by the integration of central pain modulation with controlled movement-based sensorimotor engagement, including the alleviation of transient muscle tension or protective guarding.

Future mechanistic studies incorporating neurophysiological or biomechanical assessments are warranted to further elucidate the independent and synergistic contributions of acupuncture and guided movement within the MSAT framework.

Subgroup analyses revealed a significant interaction only for baseline resting pain, where participants with lower resting pain (< 40 mm) showed greater improvement in movement-related pain following MSAT. However, as these analyses were exploratory and conducted without adjustment for multiple comparisons, the findings should be viewed strictly as hypothesis-generating and warrant confirmation in future adequately powered studies.

This study has several limitations. First, this study had a small number of dropouts (5.5%, n = 7), including participants who were lost to follow-up or discontinued the intervention. Notably, all treatment discontinuations occurred in the acupuncture group and were due to dissatisfaction with group allocation rather than rapid clinical improvement. Importantly, no dropouts were attributable to early symptom recovery, a common source of bias in acute pain studies. Despite these discontinuations, sensitivity analyses using the last observation carried forward method yielded results consistent with the primary analysis, indicating that the impact of dropouts on the overall findings was minimal. Second, the acupoints and needling techniques used in the MSAT and traditional acupuncture groups may have differed slightly, particularly due to the requirement to facilitate movement during MSAT. As a result, minor procedural variations, such as differences in insertion angle or depth, could have occurred. However, most acupoints overlapped between the two groups (Supplementary Table 2), supporting the interpretation of MSAT as an additive rather than distinct intervention. Nevertheless, parameters such as needle depth and angle of insertion were not systematically recorded, which limits direct comparison of needling techniques between groups. Third, because a sham or minimal acupuncture control was not employed, this study was not designed to distinguish the specific physiological effects of acupuncture from non-specific contextual effects, such as patient expectations. Instead, the findings should be interpreted as reflecting the incremental benefit of integrating guided movement into conventional acupuncture within a pragmatic clinical framework. In addition, this study was not designed to isolate the independent contributions of guided movement versus acupuncture; thus, the individual effect of each component cannot be determined and remains to be fully elucidated through future factorial study designs. Fourth, the follow-up duration was limited to 8 weeks, which was insufficient to assess the potential role of MSAT in preventing progression to chronic neck pain. While the present study demonstrated earlier recovery during the acute phase with MSAT compared with acupuncture, its long-term impact on pain chronicity cannot be inferred. Future large-scale trials with extended follow-up are warranted to clarify these longer-term outcomes. Fifth, interpretation of treatment response was limited by the absence of an established minimal clinically important difference (MCID) for pain intensity measured by the VAS in acute neck pain. Although the authors have previously derived an MCID estimate for VAS in acute neck pain using the present dataset [23], applying it in this context would risk circular reasoning. Therefore, the clinical relevance of the observed between-group differences should be interpreted with caution beyond statistical significance. Finally, the lack of participant and practitioner blinding, which is inherent to movement-based interventions, may have introduced performance-related bias, potentially influencing the observed treatment effects.

The safety profile of MSAT was comparable to that of conventional acupuncture, despite the incorporation of movement components during treatment. These findings indicate that MSAT can be safely implemented in clinical practice. Given its rapid effects on pain relief and functional improvement, MSAT may represent a clinically useful option for facilitating early return to daily activities in individuals with acute neck pain.

In this multicenter randomized controlled trial, MSAT was associated with more rapid improvements in pain intensity, functional status, and quality of life than conventional acupuncture in patients with acute neck pain. The integration of guided passive and active movements with acupuncture appeared to enhance early pain relief and functional recovery. Collectively, these results suggest that MSAT may be a safe and clinically meaningful therapeutic strategy within a pragmatic treatment framework for acute neck pain.

## Supplementary Information

Supplementary material 1

## Data Availability

All data are available upon reasonable request to the corresponding author.

## Abbreviations

AEs: Adverse events
ANCOVA: Analysis of covariance
AUC: Area under the curve
MSAT: Motion-style acupuncture treatment
NDI: Neck disability index
NPQ: Northwick Park neck pain questionnaire
NRS: Numerical rating scale
PCS: Physical component score
ROM: Range of motion
EQ-5D-5L: EuroQol 5-dimension 5-level questionnaire
SF-12: 12-Item short form survey
VAS: Visual analog scale
