# Supplementary material for: Effectiveness and safety of motion style acupuncture treatment for acute neck pain: a multicenter randomized controlled trial
Source: Chin Med. 2026 Jan 23;21:49. doi: 10.1186/s13020-026-01332-0 (PMC12829263; doi:10.1186/s13020-026-01332-0)
Supplement: Supplementary file 1 — Supplementary material 1 [file 13020_2026_1332_MOESM1_ESM.docx]

Supplementary Table 1 Study Visit Schedule

| **Time Point** | **Study Period** | | | | | |
| --- | --- | --- | --- | --- | --- | --- |
|  | Screening | Enroll-ment | Intervention | | Follow up | |
|  | Week -1  (V1) | Week 0  (V2) | Week 1  (V2-4) | Week 2  (V5-7) | Week 3  (V8) | Week 9  (V9) |
| Window period^1^ | -7 | Control point |  | ±3 | ±3 | ±7 |
| **Enrollment** |  |  |  |  |  |  |
| Eligibility screening | O |  |  |  |  |  |
| Written Informed consent | O |  |  |  |  |  |
| Vital signs | O | O | O (V2,4) | O (V7) | O | O |
| Sociodemographic characteristics | O |  |  |  |  |  |
| medical history | O |  |  |  |  |  |
| C-spine X-ray | O |  |  |  |  |  |
| Randomized allocation |  | O |  |  |  |  |
| **Interventions** |  |  |  |  |  |  |
| MSAT (experimental) |  |  | ←3(2~3)times/wk→ | |  |  |
| Acupuncture  (active control) |  |  | ←3(2~3)times/wk→ | |  |  |
| **Assessments** |  |  |  |  |  |  |
| Physical examination & ROM |  | O | O (V2,4) | O (V7) | O | O |
| Drug Consumption | O | O | ← every visit → | | O | O |
| Adverse events |  | O | ← every visit → | | O | O |
| VAS of Neck pain^2^ | O |  | O (V2,4) | O (V7) | O | O |
| NRS of Neck pain^2^ |  |  | O (V2,4) | O (V7) | O | O |
| NPQ |  |  | O (V2) |  | O | O |
| NDI |  |  | O (V2,4) | O (V7) | O | O |
| SF-12 |  |  | O (V2) |  | O | O |
| EQ-5D-5L |  |  | O (V2) |  | O | O |
| EQ-VAS |  |  | O (V2) |  | O | O |
| PGIC |  |  |  |  | O | O |
| Healthcare costs |  |  | ← every visit → | | O | O |
| Non-healthcare costs |  |  | O (V4) |  |  |  |
| Loss of productivity |  |  | O (V4) | O (V7) | O | O |
| Compliance |  |  |  |  |  | O |
| Credibility and Expectancy |  | O |  |  |  |  |

***MSAT***, Motion Style Acupuncture Treatment; **AT**, Acupuncture Treatment; ***VAS***, Visual Analog Scale; ***NRS***, Numeric Rating Scale; ***EQ-5D***-***5L,*** EuroQol 5-Dimension 5-level; ***SF-12,*** 12-item Short-Form Health Survey; ***PCS,*** Physical Component Summary; ***MCS***, Mental Component Summary; ***PGIC***, Patient Global Impression of Change; ***EQ-VAS***, EuroQol-5 dimension visual analog scale; ***NDI,*** Neck Disability Index; ***NPQ***, Northwick Park Neck Pain Questionnaire; ***wk***, week; ***V***, visit

**Supplementary Table 2. Baseline Characteristics of Participants by Randomized Group**

|  | MSAT (n = 64) | Acupuncture (n = 64) | P Value |  |
| --- | --- | --- | --- | --- |
| Pain location |  |  |  |  |
| Neck | 59 (92.2) | 60 (93.8) | 1.00 |  |
| Upper trapezius | 56 (87.5) | 55 (85.9) | 1.00 |  |
| Medial scapular region | 25 (87.5) | 24 (37.5) | 1.00 |  |
| Other | 7 (10.9) | 6 (9.4) | 1.00 |  |
| Current pain characteristics | | | |  |
| Right | 4 (6.2) | 5 (7.8) | 0.813 |  |
| Left | 12 (18.8) | 9 (14.1) |  |  |
| Both | 48 (75.0) | 50 (78.1) |  |  |
| Initial onset characteristics | | | |  |
| Gradual onset during daily activities | 47 (73.4) | 52 (81.2) | 0.399 |  |
| Sudden onset | 16 (25.0) | 12 (18.8) |  |  |
| Uncertain | 1 (1.6) | 0 (0.0) |  |  |
| Aggravating movements | | | |  |
| Flexion | 38 (59.4) | 38 (59.4) | 1 |  |
| Extension | 47 (73.4) | 43 (67.2) | 0.562 |  |
| Rotation | 37 (57.8) | 43 (67.2) | 0.361 |  |
| Lateral flexion | 28 (43.8) | 29 (45.3) | 1 |  |
| Aggravating circumstances | | | |  |
| Psychological stress | 30 (46.9) | 26 (40.6) | 0.593 |  |
| Fatigue | 32 (50.0) | 34 (53.1) | 0.86 |  |
| Physical work | 45 (70.3) | 36 (56.2) | 0.142 |  |
| Previous episodes of neck pain | 33 (51.6) | 38 (59.4) | 0.477 |  |
| Sensation during flexion | | | |  |
| None | 13 (20.3) | 14 (21.9) | 0.728 |  |
| Pain | 18 (28.1) | 16 (25.0) |  |  |
| Discomfort | 27 (42.2) | 31 (48.4) |  |  |
| Pain and discomfort | 6 (9.4) | 3 (4.7) |  |  |
| Sensation during extension | | | |  |
| None | 10 (15.6) | 7 (10.9) | 0.458 |  |
| Pain | 30 (46.9) | 27 (42.2) |  |  |
| Discomfort | 19 (29.7) | 27 (42.2) |  |  |
| Pain and discomfort | 5 (7.8) | 3 (4.7) |  |  |
| Sensation during left lateral flexion | | | |  |
| None | 19 (29.7) | 22 (34.4) | 0.363 |  |
| Pain | 21 (32.8) | 15 (23.4) |  |  |
| Discomfort | 21 (32.8) | 15 (23.4) |  |  |
| Pain and discomfort | 5 (7.8) | 2 (3.1) |  |  |
| Sensation during right lateral flexion | | | |  |
| None | 20 (31.2) | 19 (29.7) | 0.241 |  |
| Pain | 20 (31.2) | 13 (20.3) |  |  |
| Discomfort | 20 (31.2) | 30 (46.9) |  |  |
| Pain and discomfort |  |  |  |  |
| Sensation during left rotation | | | | |
| None | | 12 (18.8) | 21 (32.8) | 0.092 |
| Pain | | 10 (15.6) | 14 (21.9) |  |
| Discomfort | | 41 (64.1) | 28 (43.8) |  |
| Pain and discomfort | | 1 (1.6) | 1 (1.6) |  |
| Sensation during right rotation | | | | |
| None | | 15 (23.4) | 27 (42.2) | 0.089 |
| Pain | | 16 (25.0) | 10 (15.6) |  |
| Discomfort | | 32 (50.0) | 25 (39.1) |  |
| Pain and discomfort | | 1 (1.6) | 2 (3.1) |  |

***MSAT***, Motion Style Acupuncture Treatment;

Supplementary Table 3 Frequency of Treatment Sessions and Acupoint Usage

|  |  | MSAT | | Control | |
| --- | --- | --- | --- | --- | --- |
| Type of acupoint | Acupoint | n (%) | mean ± SD | n (%) | mean ± SD |
| Mandatory | TE15 | 64 (1.0) | 5.5 ± 0.8 | 64 (1.0) | 5.5 ± 1.0 |
| Mandatory | SI15 | 64 (1.0) | 5.5 ± 0.8 | 64 (1.0) | 5.5 ± 1.0 |
| Mandatory | LI16 | 64 (1.0) | 5.5 ± 0.8 | 64 (1.0) | 5.5 ± 1.0 |
| Optional | GB20 | 28 (0.4) | 5.5 ± 0.8 | 30 (0.5) | 5.7 ± 0.7 |
| Optional | BL10 | 34 (0.5) | 5.7 ± 0.7 | 34 (0.5) | 5.4 ± 1.2 |
| Optional | GV14 | 9 (0.1) | 5.1 ± 1.1 | 13 (0.2) | 5.0 ± 1.7 |
| Optional | SI14 | 16 (0.2) | 5.7 ± 0.8 | 18 (0.3) | 5.1 ± 1.7 |
| Optional | EX-B2 (C5/6) | 28 (0.4) | 5.2 ± 1.1 | 24 (0.4) | 5.5 ± 0.7 |
| Optional | EX-B2 (C4/5) | 24 (0.4) | 4.8 ± 1.7 | 22 (0.3) | 5.1 ± 1.5 |
| Optional | EX-B2 (C3/4) | 7 (0.1) | 5.0 ± 1.5 | 7 (0.1) | 5.1 ± 1.6 |

***MSAT***, Motion Style Acupuncture Treatment; ***SD***, Standard Deviation

Supplementary Table 4 Range of motion outcomes by Treatment and Time since Randomization (ANCOVA)

| Assessment | Categories | Week 1 | Week 2 | Week 3 | Week 9 |
| --- | --- | --- | --- | --- | --- |
| ROM of flexion | MSAT | 42.83  (41.67 to 43.98) | 44.29  (43.39 to 45.18) | 44.63  (43.81 to 45.45) | 44.24  (43.47 to 45.02) |
|  | AT | 42.15  (40.99 to 43.31) | 42.81  (41.91 to 43.71) | 43.14  (42.31 to 43.98) | 44.39  (43.62 to 45.16) |
|  | Difference | -0.67  (-2.31 to 0.96) | -1.48  (-2.74 to -0.21) | -1.48  (-2.66 to -0.31) | 0.15  (-0.94 to 1.23) |
|  | *P*-value | 0.415 | 0.023* | 0.014* | 0.788 |
| ROM of extension | MSAT | 42.57  (41.53 to 43.61) | 44.32  (43.55 to 45.10) | 44.57  (43.75 to 45.39) | 44.79  (44.44 to 45.14) |
|  | AT | 43.13  (42.08 to 44.17) | 43.72  (42.94 to 44.49) | 43.90  (43.07 to 44.74) | 44.82  (44.47 to 45.17) |
|  | Difference | 0.56  (-0.91 to 2.03) | -0.61  (-1.70 to 0.49) | -0.67  (-1.84 to 0.51) | 0.03 (-0.46 to 0.53) |
|  | *P*-value | 0.455 | 0.274 | 0.263 | 0.899 |
| ROM of Left lateral flexion | MSAT | 42.37  (40.97 to 43.77) | 43.19  (41.90 to 44.48) | 43.59  (42.49 to 44.69) | 43.89  (42.88 to 44.90) |
|  | AT | 40.73  (39.30 to 42.16) | 42.51  (41.19 to 43.83) | 43.35  (42.22 to 44.48) | 43.79  (42.77 to 44.80) |
|  | Difference | -1.64  (-3.65 to 0.36) | -0.68  (-2.53 to 1.17) | -0.24  (-1.82 to 1.34) | -0.10  (-1.53 to 1.32) |
|  | *P*-value | 0.107 | 0.47 | 0.767 | 0.887 |
| ROM of Right lateral flexion | MSAT | 41.64  (40.24 to 43.05) | 42.93  (41.64 to 44.21) | 43.78  (42.58 to 44.97) | 43.98  (42.96 to 45.00) |
|  | AT | 40.10  (38.66 to 41.55) | 42.46  (41.15 to 43.78) | 42.84  (41.62 to 44.05) | 43.30  (42.28 to 44.33) |
|  | Difference | -1.54  (-3.55 to 0.48) | -0.47  (-2.31 to 1.37) | -0.94  (-2.65 to 0.76) | -0.68  (-2.12 to 0.76) |
|  | *P*-value | 0.134 | 0.617 | 0.277 | 0.351 |
| ROM of Left rotation | MSAT | 84.11  (81.86 to 86.36) | 86.11  (84.21 to 88.01) | 87.84  (86.66 to 89.02) | 88.31  (86.95 to 89.68) |
|  | AT | 84.19  (81.90 to 86.48) | 85.90  (83.92 to 87.88) | 88.35  (87.13 to 89.58) | 88.97  (87.62 to 90.32) |
|  | Difference | 0.08  (-3.12 to 3.29) | -0.21  (-2.95 to 2.54) | 0.51  (-1.19 to 2.22) | 0.66  (-1.27 to 2.58) |
|  | *P*-value | 0.96 | 0.882 | 0.553 | 0.5 |
| ROM of Right rotation | MSAT | 85.01  (82.77 to 87.25) | 87.30  (85.66 to 88.93) | 88.09  (86.63 to 89.55) | 88.19  (86.83 to 89.55) |
|  | AT | 83.81  (81.51 to 86.10) | 86.07  (84.41 to 87.73) | 87.43  (85.93 to 88.94) | 89.06  (87.73 to 90.39) |
|  | Difference | -1.20  (-4.41 to 2.02) | -1.23  (-3.56 to 1.11) | -0.65  (-2.76 to 1.45) | 0.87 (-1.03 to 2.78) |
|  | *P*-value | 0.462 | 0.301 | 0.539 | 0.367 |
| WPAI | MSAT | 46.36  (42.08 to 50.65) | 30.30  (25.80 to 34.80) | 23.22  (18.76 to 27.68) | 17.25  (12.53 to 21.98) |
|  | AT | 50.25  (45.83 to 54.67) | 30.30  (25.80 to 34.80) | 39.90  (35.42 to 44.37) | 30.43  (25.70 to 35.16) |
|  | Difference | -3.88  (-10.04 to 2.27) | -8.79  (-15.22 to -2.36) | -16.68  (-23.01 to -10.35) | -13.18  (-19.87 to -6.49) |
|  | *P*-value | 0.214 | 0.008** | <0.001*** | <0.001*** |

*MSAT*, Motion Style Acupuncture Treatment; AT, Acupuncture Treatment; *ROM*, Range of Movement

Supplementary Table 5 Outcomes by Treatment and Time since Randomization (LMM)

| Assessment | Categories | Week 1 | Week 2 | Week 3 | Week 9 |
| --- | --- | --- | --- | --- | --- |
| VAS on movement | MSAT | 46.08  (42.21 to 49.95) | 29.05  (25.17 to 32.92) | 22.71  (18.81 to 26.62) | 17.88  (13.96 to 21.80) |
|  | AT | 53.83  (49.90 to 57.77) | 43.74  (39.80 to 47.67) | 38.71  (34.76 to 42.66) | 33.50  (29.55 to 37.45) |
|  | Difference | 7.75  (2.22 to 13.29) | 14.69  (9.15 to 20.23) | 15.99  (10.42 to 21.56) | 15.61  (10.03 to 21.20) |
|  | *P*-value | 0.006** | 14.69  (9.15 to 20.23) | <0.001*** | <0.001*** |
| VAS at rest | MSAT | 39.37  (35.70 to 43.04) | 23.27  (19.60 to 26.95) | 17.63  (13.93 to 21.34) | 13.41  (9.69 to 17.14) |
|  | AT | 43.96  (40.23 to 47.69) | 34.42  (30.69 to 38.16) | 30.75  (27.00 to 34.50) | 26.48  (22.73 to 30.23) |
|  | Difference | 4.59  (-0.65 to 9.82) | 11.15  (5.92 to 16.39) | 13.11  (7.84 to 18.39) | 13.07  (7.78 to 18.35) |
|  | *P*-value | 0.086 | <0.001*** | <0.001*** | <0.001*** |
| NRS on movement | MSAT | 4.64  (4.26 to 5.02) | 2.94  (2.56 to 3.32) | 2.29 (1.90 to 2.67) | 1.77 (1.38 to 2.16) |
|  | AT | 5.31  (4.93 to 5.70) | 4.28  (3.89 to 4.67) | 3.74 (3.35 to 4.13) | 3.23 (2.84 to 3.62) |
|  | Difference | 0.67  (0.13 to 1.22) | 4.28  (3.89 to 4.67) | 1.45 (0.90 to 2.00) | 1.46 (0.91 to 2.01) |
|  | *P*-value | 0.016* | <0.001*** | <0.001*** | <0.001*** |
| NRS at rest | MSAT | 3.95  (3.58 to 4.31) | 2.34  (1.97 to 2.70) | 1.68  (1.31 to 2.04) | 1.27  (0.90 to 1.63) |
|  | AT | 4.37  (4.00 to 4.74) | 3.45  (3.08 to 3.82) | 3.08  (2.71 to 3.46) | 2.58  (2.21 to 2.95) |
|  | Difference | 0.42  (-0.10 to 0.94) | 1.11  ( 0.59 to 1.63) | 1.41  ( 0.88 to 1.93) | 1.32  ( 0.79 to 1.84) |
|  | *P*-value | 0.11 | <0.001*** | <0.001*** | <0.001*** |
| NDI | MSAT | 22.88  (20.68 to 25.09) | 15.16  (12.96 to 17.36) | 14.27  (12.05 to 16.49) | 13.38  (11.15 to 15.61) |
|  | AT | 24.53  (22.29 to 26.77) | 20.66  (18.42 to 22.89) | 22.14  (19.89 to 24.39) | 18.66  (16.42 to 20.91) |
|  | Difference | 1.65  (-1.50 to 4.79) | 5.50  ( 2.36 to 8.64) | 7.87  (4.71 to 11.03) | 5.29  (2.12 to 8.45) |
|  | *P*-value | 0.303 | <0.001*** | <0.001*** | 0.001** |
| NPQ | MSAT | - | - | 18.89  (15.99 to 21.79) | 17.13  (14.22 to 20.04) |
|  | AT | - | - | 26.51  (23.60 to 29.42) | 23.59  (20.68 to 26.51) |
|  | Difference | - | - | 7.62  (3.50 to 11.74) | 6.46  (2.33 to 10.60) |
|  | *P*-value | - | - | <0.001*** | 0.002** |
| EQ5D | MSAT | - | - | 0.84  (0.82 to 0.86) | 0.85  (0.83 to 0.87) |
|  | AT | - | - | 0.82  (0.80 to 0.84) | 0.82  (0.80 to 0.84) |
|  | Difference | - | - | -0.02  (-0.05 to 0.01) | -0.03  (-0.06 to 0.00) |
|  | *P*-value | - | - | 0.108 | 0.035* |
| EQVAS | MSAT | - | - | 75.00  (71.26 to 78.73) | 77.27  (73.52 to 81.03) |
|  | AT | - | - | 67.87  (64.12 to 71.63) | 75.39  (71.63 to 79.15) |
|  | Difference | - | - | -7.12  (-12.42 to -1.82) | -1.88  ( -7.20 to 3.43) |
|  | *P*-value | - | - | 0.009** | 0.486 |
| PCS  (SF-12) | MSAT | - | - | 48.96  (47.41 to 50.52) | 50.16  (48.60 to 51.73) |
|  | AT | - | - | 46.23  (44.67 to 47.79) | 48.51  (46.95 to 50.07) |
|  | Difference | - | - | -2.73  (-4.94 to -0.53) | -1.65  (-3.87 to 0.56) |
|  | *P*-value | - | - | 0.015* | 0.142 |
| MCS  (SF-12) | MSAT | - | - | 50.75  (48.86 to 52.63) | 51.28  (49.38 to 53.17) |
|  | AT | - | - | 49.83  (47.93 to 51.73) | 51.14  (49.24 to 53.04) |
|  | Difference | - | - | -0.92  (-3.59 to 1.76) | -0.14  (-2.82 to 2.55) |
|  | *P*-value | - | - | 0.5 | 0.92 |
| ROM of flexion | MSAT | 42.85  (41.91 to 43.80) | 44.34  (43.39 to 45.28) | 44.76  (43.80 to 45.72) | 44.45  (43.48 to 45.41) |
|  | AT | 42.26  (41.30 to 43.22) | 42.90  (41.94 to 43.87) | 43.22  (42.26 to 44.19) | 44.46  (43.49 to 45.43) |
|  | Difference | -0.59  (-1.94 to 0.76) | -1.43  (-2.78 to -0.08) | -1.54  (-2.90 to -0.17) | 0.01  (-1.35 to 1.38) |
|  | *P*-value | 0.389 | 0.038* | 0.027* | 0.987 |
| ROM of extension | MSAT | 42.58  (41.76 to 43.39) | 44.37  (43.56 to 45.18) | 44.70  (43.87 to 45.52) | 44.91  (44.08 to 45.75) |
|  | AT | 43.24  (42.42 to 44.07) | 43.81  (42.98 to 44.63) | 43.96  (43.13 to 44.79) | 44.86  (44.03 to 45.70) |
|  | Difference | 0.67  (-0.49 to 1.82) | -0.57  (-1.72 to 0.59) | -0.74  (-1.91 to 0.43) | -0.05  (-1.23 to 1.13) |
|  | *P*-value | 0.259 | 0.337 | 0.217 | 0.933 |
| ROM of Left lateral flexion | MSAT | 42.18  (40.95 to 43.42) | 43.12  (41.89 to 44.35) | 43.61  (42.36 to 44.86) | 43.95  (42.69 to 45.21) |
|  | AT | 40.75  (39.50 to 42.00) | 42.36  (41.11 to 43.62) | 43.16  (41.90 to 44.42) | 43.57  (42.30 to 44.83) |
|  | Difference | -1.43  (-3.19 to 0.33) | -0.76  (-2.52 to 1.00) | -0.45  (-2.23 to 1.33) | -0.38  (-2.17 to 1.40) |
|  | *P*-value | 0.11 | 0.398 | 0.618 | 0.673 |
| ROM of Right lateral flexion | MSAT | 41.51  (40.25 to 42.78) | 42.84  (41.58 to 44.11) | 43.74  (42.46 to 45.02) | 43.98  (42.69 to 45.27) |
|  | AT | 40.03  (38.74 to 41.31) | 42.29  (41.00 to 43.57) | 42.67  (41.38 to 43.96) | 43.09  (41.80 to 44.38) |
|  | Difference | -1.49  (-3.29 to 0.32) | -0.56  (-2.36 to 1.25) | -1.07  (-2.89 to 0.75) | -0.89  (-2.71 to 0.93) |
|  | *P*-value | 0.106 | 0.545 | 0.249 | 0.337 |
| ROM of Left rotation | MSAT | 84.21  (82.41 to 86.00) | 86.24  (84.44 to 88.03) | 88.07  (86.25 to 89.89) | 88.47  (86.64 to 90.31) |
|  | AT | 84.43  (82.61 to 86.26) | 86.21  (84.39 to 88.03) | 88.64  (86.80 to 90.47) | 89.14  (87.31 to 90.98) |
|  | Difference | 0.23  (-2.33 to 2.78) | -0.03  (-2.59 to 2.53) | 0.56  (-2.02 to 3.15) | 0.67  (-1.93 to 3.27) |
|  | *P*-value | 0.862 | 0.981 | 0.669 | 0.612 |
| ROM of Right rotation | MSAT | 84.90  (83.16 to 86.64) | 87.40  (85.66 to 89.14) | 88.31  (86.54 to 90.07) | 88.51  (86.74 to 90.28) |
|  | AT | 84.24  (82.48 to 86.01) | 86.26  (84.49 to 88.03) | 87.68  (85.91 to 89.46) | 89.04  (87.26 to 90.82) |
|  | Difference | -0.65  (-3.14 to 1.83) | -1.14  (-3.62 to 1.34) | -0.62  (-3.13 to 1.88) | 0.53  (-1.98 to 3.05) |
|  | *P*-value | 0.604 | 0.368 | 0.626 | 0.676 |
| WPAI | MSAT | 46.36  (41.92 to 50.81) | 30.30  (25.85 to 34.75) | 22.90  (18.42 to 27.39) | 16.54  (12.03 to 21.05) |
|  | AT | 50.30  (45.78 to 54.81) | 39.36  (34.84 to 43.88) | 40.17  (35.63 to 44.71) | 30.85  (26.29 to 35.41) |
|  | Difference | -3.93  (-10.27 to 2.41) | -9.06  (-15.40 to -2.71) | -17.26  (-23.65 to -10.88) | -14.31  (-20.72 to -7.90) |
|  | *P*-value | 0.223 | 0.005** | <0.001*** | <0.001*** |
| PGIC | MSAT | - | - | 2.00  (1.78 to 2.22) | 2.19  (1.97 to 2.41) |
|  | AT | - | - | 2.80  (2.58 to 3.02) | 2.62  (2.40 to 2.85) |
|  | Difference | - | - | -0.80  (-1.11 to -0.49) | -0.44  (-0.75 to -0.12) |
|  | *P*-value | - | - | <0.001*** | 0.006** |

***MSAT***, Motion Style Acupuncture Treatment; **AT**, Acupuncture Treatment; ***VAS***, Visual Analog Scale; ***NRS***, Numeric Rating Scale; ***EQ-5D***-***5L,*** EuroQol 5-Dimension 5-level; ***SF-12,*** 12-item Short-Form Health Survey; ***PCS,*** Physical Component Summary; ***MCS***, Mental Component Summary; ***PGIC***, Patient Global Impression of Change; ***EQ-VAS***, EuroQol-5 dimension visual analog scale; ***NDI***: Neck Disability Index; ***NPQ***: Northwick Park Neck Pain Questionnaire; ***WPAI***; Work Productivity and Activity Impairment Questionnaire

Supplementary Table 6 Outcomes by Treatment and Time since Randomization (LOCF)

| Assessment | Categories | Week 1 | Week 2 | Week 3 | Week 9 |
| --- | --- | --- | --- | --- | --- |
| VAS on movement | MSAT | 45.89  (42.78 to 49.01) | 28.85  (24.95 to 32.74) | 22.74  (18.57 to 26.92) | 18.30  (13.78 to 22.82) |
|  | AT | 54.40  (51.29 to 57.51) | 44.64  (40.74 to 48.53) | 39.85  (35.68 to 44.02) | 34.56  (30.04 to 39.08) |
|  | Difference | 8.51  (4.10 to 12.92) | 15.79  (10.26 to 21.31) | 17.10  (11.19 to 23.02) | 16.26  (9.85 to 22.67) |
|  | *P*-value | <0.001*** | <0.001*** | <0.001*** | <0.001*** |
| VAS at rest | MSAT | 39.43  (36.54 to 42.31) | 23.36  (19.89 to 26.83) | 18.01  (14.18 to 21.84) | 14.04  (9.73 to 18.35) |
|  | AT | 44.34  (41.45 to 47.22) | 35.08  (31.61 to 38.55) | 31.62  (27.79 to 35.44) | 27.38  (23.07 to 31.69) |
|  | Difference | 4.91  (0.83 to 8.99) | 11.72  (6.81 to 16.62) | 13.61  (8.19 to 19.02) | 13.34  (7.24 to 19.44) |
|  | *P*-value | 0.019* | <0.001*** | <0.001*** | <0.001*** |
| NRS on movement | MSAT | 4.63  (4.31 to 4.94) | 2.93  (2.55 to 3.31) | 2.30  (1.89 to 2.72) | 1.81  (1.37 to 2.26) |
|  | AT | 5.37  (5.06 to 5.69) | 4.37  (3.99 to 4.75) | 3.85  (3.44 to 4.26) | 3.34  (2.90 to 3.79) |
|  | Difference | 0.75  (0.30 to 1.19) | 1.44  (0.90 to 1.98) | 1.55  (0.96 to 2.13) | 1.53  (0.90 to 2.16) |
|  | *P*-value | 0.001** | <0.001*** | <0.001*** | <0.001*** |
| NRS at rest | MSAT | 3.95  (3.68 to 4.22) | 2.35  (2.00 to 2.69) | 1.73  (1.35 to 2.11) | 1.35  (0.91 to 1.78) |
|  | AT | 4.41  (4.14 to 4.68) | 3.51  (3.17 to 3.86) | 3.16  (2.79 to 3.54) | 2.67  (2.24 to 3.10) |
|  | Difference | 0.47  (0.08 to 0.85) | 1.17  (0.68 to 1.65) | 1.44  (0.90 to 1.97) | 1.33  (0.71 to 1.94) |
|  | *P*-value | 0.018* | <0.001*** | <0.001*** | <0.001*** |
| NDI | MSAT | 22.86  (20.96 to 24.77) | 15.10  (13.00 to 17.19) | 14.30  (11.94 to 16.66) | 13.43  (10.78 to 16.08) |
|  | AT | 24.92  (23.01 to 26.82) | 21.21  (19.12 to 23.30) | 22.67  (20.31 to 25.03) | 19.31  (16.65 to 21.96) |
|  | Difference | 2.06  (-0.64 to 4.75) | 6.11  (3.15 to 9.07) | 8.38  (5.04 to 11.71) | 5.88  (2.12 to 9.63) |
|  | *P*-value | 0.133 | <0.001*** | <0.001*** | 0.002** |
| NPQ | MSAT | - | - | 19.36  (16.72 to 21.99) | 17.81  (14.59 to 21.02) |
|  | AT | - | - | 27.21  (24.57 to 29.84) | 24.19  (20.97 to 27.41) |
|  | Difference | - | - | 7.85  (4.11 to 11.59) | 6.38  (1.82 to 10.95) |
|  | *P*-value | - | - | <0.001*** | 0.007** |
| EQ5D | MSAT | - | - | 0.84  (0.82 to 0.86) | 0.85  (0.83 to 0.87) |
|  | AT | - | - | 0.81  (0.79 to 0.83) | 0.82  (0.80 to 0.84) |
|  | Difference | - | - | -0.03  (-0.05 to 0.00) | -0.03  (-0.06 to 0.00) |
|  | *P*-value | - | - | 0.06 | 0.034* |
| EQVAS | MSAT | - | - | 74.81  (71.10 to 78.52) | 77.08  (73.54 to 80.63) |
|  | AT | - | - | 67.71  (63.99 to 71.42) | 75.10  (71.56 to 78.65) |
|  | Difference | - | - | -7.10  (-12.36 to -1.85) | -1.98  (-7.00 to 3.04) |
|  | *P*-value | - | - | 0.008** | 0.436 |
| PCS  (SF-12) | MSAT | - | - | 48.97  (47.49 to 50.44) | 50.15  (48.57 to 51.74) |
|  | AT | - | - | 46.06  (44.59 to 47.54) | 48.18  (46.60 to 49.77) |
|  | Difference | - | - | -2.91  (-4.99 to -0.82) | -1.97  (-4.21 to 0.27) |
|  | *P*-value | - | - | 0.007** | 0.084 |
| MCS  (SF-12) | MSAT | - | - | 50.78  (48.87 to 52.68) | 51.06  (49.18 to 52.94) |
|  | AT | - | - | 49.52  (47.61 to 51.42) | 50.88  (48.99 to 52.76) |
|  | Difference | - | - | -1.26  (-3.95 to 1.44) | -0.19  (-2.85 to 2.47) |
|  | *P*-value | - | - | 0.357 | 0.889 |
| ROM of flexion | MSAT | 42.83  (41.68 to 43.97) | 44.29  (43.40 to 45.17) | 44.63  (43.82 to 45.45) | 44.27  (43.52 to 45.02) |
|  | AT | 42.17  (41.03 to 43.32) | 42.82  (41.94 to 43.71) | 43.18  (42.36 to 43.99) | 44.41  (43.66 to 45.15) |
|  | Difference | -0.65  (-2.28 to 0.97) | -1.46  (-2.72 to -0.21) | -1.45  (-2.61 to -0.30) | 0.14  (-0.92 to 1.20) |
|  | *P*-value | 0.429 | 0.023* | 0.014* | 0.797 |
| ROM of extension | MSAT | 42.57  (41.53 to 43.61) | 44.32  (43.55 to 45.09) | 44.57  (43.77 to 45.38) | 44.79  (44.44 to 45.14) |
|  | AT | 43.13  (42.10 to 44.17) | 43.72  (42.95 to 44.49) | 43.94  (43.13 to 44.75) | 44.82  (44.47 to 45.17) |
|  | Difference | 0.57  (-0.91 to 2.04) | -0.60  (-1.69 to 0.49) | -0.63  (-1.78 to 0.51) | 0.03  (-0.46 to 0.53) |
|  | *P*-value | 0.448 | 0.279 | 0.277 | 0.899 |
| ROM of Left lateral flexion | MSAT | 42.39  (40.98 to 43.81) | 43.22  (41.86 to 44.57) | 43.63  (42.43 to 44.84) | 43.94  (42.82 to 45.06) |
|  | AT | 40.42  (39.01 to 41.84) | 42.10  (40.74 to 43.45) | 42.85  (41.64 to 44.06) | 43.32  (42.20 to 44.44) |
|  | Difference | -1.97  (-3.97 to 0.03) | -1.12  (-3.04 to 0.80) | -0.78  (-2.49 to 0.93) | -0.62  (-2.21 to 0.96) |
|  | *P*-value | 0.054 | 0.251 | 0.366 | 0.438 |
| ROM of Right lateral flexion | MSAT | 41.65  (40.23 to 43.06) | 42.94  (41.58 to 44.29) | 43.79  (42.51 to 45.07) | 44.02  (42.89 to 45.14) |
|  | AT | 39.84  (38.42 to 41.25) | 42.06  (40.71 to 43.42) | 42.38  (41.10 to 43.66) | 42.86  (41.73 to 43.99) |
|  | Difference | -1.81  (-3.81 to 0.19) | -0.87  (-2.79 to 1.04) | -1.41  (-3.22 to 0.40) | -1.16  (-2.75 to 0.44) |
|  | *P*-value | 0.075 | 0.369 | 0.126 | 0.154 |
| ROM of Left rotation | MSAT | 84.11  (81.88 to 86.34) | 86.11  (84.25 to 87.97) | 87.85  (86.70 to 89.01) | 88.22  (86.86 to 89.57) |
|  | AT | 84.33  (82.10 to 86.55) | 86.08  (84.22 to 87.93) | 88.40  (87.24 to 89.55) | 88.97  (87.61 to 90.32) |
|  | Difference | 0.22  (-2.93 to 3.37) | -0.03  (-2.66 to 2.59) | 0.54  (-1.10 to 2.18) | 0.75  (-1.17 to 2.66) |
|  | *P*-value | 0.892 | 0.98 | 0.513 | 0.44 |
| ROM of Right rotation | MSAT | 85.01  (82.80 to 87.23) | 87.30  (85.67 to 88.92) | 88.09  (86.66 to 89.52) | 88.12  (86.81 to 89.44) |
|  | AT | 83.97  (81.75 to 86.19) | 86.14  (84.51 to 87.76) | 87.54  (86.10 to 88.97) | 89.06  (87.75 to 90.38) |
|  | Difference | -1.04  (-4.19 to 2.10) | -1.16  (-3.47 to 1.15) | -0.55  (-2.58 to 1.48) | 0.94  (-0.93 to 2.80) |
|  | *P*-value | 0.513 | 0.321 | 0.59 | 0.321 |
| WPAI | MSAT | 46.36  (42.13 to 50.60) | 30.30  (25.84 to 34.77) | 23.04  (18.67 to 27.41) | 16.91  (12.21 to 21.61) |
|  | AT | 46.36  (42.13 to 50.60) | 39.70  (35.24 to 44.16) | 40.54  (36.17 to 44.91) | 32.03  (27.34 to 36.73) |
|  | Difference | -3.93  (-9.93 to 2.06) | -9.40  (-15.71 to -3.09) | -17.50  (-23.68 to -11.33) | -15.12  (-21.76 to -8.48) |
|  | *P*-value | 0.197 | 0.004** | <0.001*** | <0.001*** |
| PGIC | MSAT | - | - | 2.00  (1.80 to 2.20) | 2.17  (1.94 to 2.40) |
|  | AT | - | - | 2.79  (2.59 to 2.98) | 2.63  (2.41 to 2.86) |
|  | Difference | - | - | -0.79  (-1.07 to -0.51) | -0.46  (-0.79 to -0.14) |
|  | *P*-value | - | - | <0.001*** | 0.005** |

***MSAT***, Motion Style Acupuncture Treatment; **AT**, Acupuncture Treatment; ***VAS***, Visual Analog Scale; ***NRS***, Numeric Rating Scale; ***EQ-5D***-***5L,*** EuroQol 5-Dimension 5-level; ***SF-12,*** 12-item Short-Form Health Survey; ***PCS,*** Physical Component Summary; ***MCS***, Mental Component Summary; ***PGIC***, Patient Global Impression of Change; ***EQ-VAS***, EuroQol-5 dimension visual analog scale; ***NDI***: Neck Disability Index; ***NPQ***: Northwick Park Neck Pain Questionnaire; ***WPAI***; Work Productivity and Activity Impairment Questionnaire

Supplementary Table 7 Outcomes by Treatment and Time since Randomization (Per protocol)

| Assessment | Categories | Week 1 | Week 2 | Week 3 | Week 9 |
| --- | --- | --- | --- | --- | --- |
| VAS on movement | MSAT | 46.40  (43.25 to 49.55) | 29.48  (25.58 to 33.38) | 23.08  (18.92 to 27.25) | 18.49  (13.95 to 23.04) |
|  | AT | 54.25  (51.07 to 57.42) | 44.18  (40.24 to 48.11) | 38.95  (34.73 to 43.16) | 32.90  (28.32 to 37.48) |
|  | Difference | 7.85  (3.36 to 12.34) | 14.70  (9.13 to 20.26) | 15.87  (9.92 to 21.82) | 14.40  (7.93 to 20.88) |
|  | *P*-value | <0.001*** | <0.001*** | <0.001*** | <0.001*** |
| VAS at rest | MSAT | 40.03  (37.11 to 42.94) | 23.75  (20.23 to 27.26) | 18.17  (14.28 to 22.06) | 14.06  ( 9.62 to 18.51) |
|  | AT | 44.89  (41.95 to 47.83) | 35.44  (31.89 to 38.99) | 31.57  (27.64 to 35.51) | 26.60  (22.12 to 31.09) |
|  | Difference | 4.86  (0.72 to 9.00) | 11.70  (6.70 to 16.70) | 13.40  (7.87 to 18.94) | 12.54  (6.22 to 18.86) |
|  | *P*-value | 0.022* | <0.001*** | <0.001*** | <0.001*** |
| NRS on movement | MSAT | 4.68  (4.36 to 5.00) | 2.98  (2.61 to 3.36) | 2.33  (1.92 to 2.74) | 1.82  (1.38 to 2.27) |
|  | AT | 5.36  (5.03 to 5.68) | 4.33  (3.95 to 4.71) | 3.77  (3.36 to 4.18) | 3.18  (2.73 to 3.63) |
|  | Difference | 0.67  (0.22 to 1.13) | 1.35  (0.81 to 1.89) | 1.44  (0.86 to 2.02) | 1.36  (0.72 to 1.99) |
|  | *P*-value | 0.004** | <0.001*** | <0.001*** | <0.001*** |
| NRS at rest | MSAT | 4.01  (3.73 to 4.29) | 2.38  (2.04 to 2.73) | 1.74  (1.35 to 2.12) | 1.34  (0.90 to 1.79) |
|  | AT | 4.47  (4.20 to 4.75) | 3.54  (3.19 to 3.89) | 3.15  (2.76 to 3.53) | 2.59  (2.14 to 3.03) |
|  | Difference | 0.46  (0.07 to 0.85) | 1.16  (0.66 to 1.65) | 1.41  (0.86 to 1.96) | 1.24  (0.61 to 1.88) |
|  | *P*-value | 0.021* | <0.001*** | <0.001*** | <0.001*** |
| NDI | MSAT | 23.05  (21.15 to 24.95) | 15.14  (13.08 to 17.20) | 14.32  (11.95 to 16.69) | 13.42  (10.74 to 16.11) |
|  | AT | 24.73  (22.82 to 26.64) | 20.73  (18.66 to 22.81) | 22.23  (19.83 to 24.63) | 18.67  (15.97 to 21.37) |
|  | Difference | 1.68  (-1.01 to 4.38) | 5.60  (2.67 to 8.52) | 7.91  (4.54 to 11.28) | 5.25  (1.44 to 9.05) |
|  | *P*-value | 0.219 | <0.001*** | <0.001*** | 0.007** |
| NPQ | MSAT | - | - | 18.90  (16.31 to 21.49) | 17.25  (14.00 to 20.50) |
|  | AT | - | - | 26.61  (24.00 to 29.23) | 23.46  (20.18 to 26.74) |
|  | Difference | - | - | 7.72  (4.02 to 11.41) | 6.21  (1.58 to 10.85) |
|  | *P*-value | - | - | <0.001*** | 0.009** |
| EQ5D | MSAT | - | - | 0.84  (0.82 to 0.86) | 0.86  (0.83 to 0.88) |
|  | AT | - | - | 0.81  (0.79 to 0.83) | 0.82  (0.80 to 0.84) |
|  | Difference | - | - | -0.03  (-0.06 to 0.00) | -0.03  (-0.06 to 0.00) |
|  | *P*-value | - | - | 0.034* | 0.401 |
| EQVAS | MSAT | - | - | 74.75  (70.95 to 78.56) | 77.13  (73.47 to 80.79) |
|  | AT | - | - | 67.28  (63.43 to 71.14) | 74.92  (71.23 to 78.61) |
|  | Difference | - | - | -7.47  (-12.89 to -2.06) | -2.21  (-7.41 to 2.99) |
|  | *P*-value | - | - | 0.007** | 0.401 |
| PCS  (SF-12) | MSAT | - | - | 48.84  (47.33 to 50.34) | 50.10  (48.48 to 51.73) |
|  | AT | - | - | 46.53  (45.00 to 48.06) | 48.64  (47.00 to 50.27) |
|  | Difference | - | - | -2.31  (-4.46 to -0.16) | -1.47  (-3.78 to 0.84) |
|  | *P*-value | - | - | 0.036* | 0.211 |
| MCS  (SF-12) | MSAT | - | - | 51.17  (49.31 to 53.02) | 51.46  (49.63 to 53.30) |
|  | AT | - | - | 49.45  (47.57 to 51.34) | 51.00  (49.15 to 52.86) |
|  | Difference | - | - | -1.71  (-4.35 to 0.93) | -0.46  (-3.06 to 2.15) |
|  | *P*-value | - | - | 0.202 | 0.729 |
| ROM of flexion | MSAT | 42.72  (41.51 to 43.93) | 44.25  (43.32 to 45.19) | 44.62  (43.76 to 45.47) | 44.23  (43.45 to 45.02) |
|  | AT | 42.07  (40.85 to 43.29) | 42.76  (41.82 to 43.70) | 43.04  (42.18 to 43.91) | 44.45  (43.66 to 45.24) |
|  | Difference | -0.65  (-2.38 to 1.07) | -1.50  (-2.82 to -0.17) | -1.57  (-2.79 to -0.35) | 0.22  (-0.90 to 1.33) |
|  | *P*-value | 0.453 | 0.028* | 0.012* | 0.7 |
| ROM of extension | MSAT | 42.41  (41.33 to 43.50) | 44.26  (43.46 to 45.07) | 44.54  (43.68 to 45.40) | 44.77  (44.42 to 45.12) |
|  | AT | 43.13  (42.04 to 44.22) | 43.75  (42.94 to 44.56) | 43.85  (42.98 to 44.73) | 44.90  (44.54 to 45.25) |
|  | Difference | 0.72  (-0.82 to 2.26) | -0.52  (-1.66 to 0.63) | -0.69  (-1.92 to 0.54) | 0.13  (-0.37 to 0.63) |
|  | *P*-value | 0.359 | 0.373 | 0.269 | 0.62 |
| ROM of Left lateral flexion | MSAT | 42.41  (40.96 to 43.85) | 43.19  (41.84 to 44.54) | 43.59  (42.45 to 44.73) | 43.92  (42.88 to 44.95) |
|  | AT | 40.47  (39.01 to 41.93) | 42.34  (40.98 to 43.70) | 43.23  (42.06 to 44.41) | 43.69  (42.64 to 44.73) |
|  | Difference | 40.47  (39.01 to 41.93) | -0.85  (-2.77 to 1.07) | -0.36  (-2.00 to 1.28) | -0.23  (-1.71 to 1.25) |
|  | *P*-value | 0.065 | 0.382 | 0.669 | 0.759 |
| ROM of Right lateral flexion | MSAT | 41.58  (40.15 to 43.02) | 42.88  (41.54 to 44.21) | 43.77  (42.52 to 45.01) | 43.99  (42.94 to 45.03) |
|  | AT | 39.89  (38.45 to 41.34) | 42.32  (40.98 to 43.67) | 42.69  (41.42 to 43.96) | 43.20  (42.14 to 44.25) |
|  | Difference | -1.69  (-3.73 to 0.35) | -0.55  (-2.45 to 1.35) | -1.08  (-2.86 to 0.70) | -0.79  (-2.28 to 0.69) |
|  | *P*-value | 0.103 | 0.565 | 0.233 | 0.293 |
| ROM of Left rotation | MSAT | 84.04  (81.69 to 86.38) | 86.16  (84.22 to 88.11) | 87.88  (86.64 to 89.11) | 88.28  (86.86 to 89.70) |
|  | AT | 84.15  (81.78 to 86.51) | 85.90  (83.94 to 87.86) | 88.31  (87.04 to 89.58) | 88.91  (87.49 to 90.34) |
|  | Difference | 0.11 (-3.22 to 3.44) | -0.26  (-3.02 to 2.50) | 0.44  (-1.34 to 2.21) | 0.63  (-1.38 to 2.65) |
|  | *P*-value | 0.948 | 0.852 | 0.628 | 0.534 |
| ROM of Right rotation | MSAT | 84.97  (82.64 to 87.30) | 87.40  (85.71 to 89.10) |  |  |
|  | AT | 83.78  (81.43 to 86.13) | 85.97  (84.27 to 87.68) |  |  |
|  | Difference | -1.19  (-4.51 to 2.13) | -1.43  (-3.84 to 0.98) |  |  |
|  | *P*-value | 0.48 | 0.242 |  |  |
| WPAI | MSAT | 47.20  (42.81 to 51.60) | 31.30  (26.73 to 35.87) | 23.68  (19.20 to 28.15) | 17.25  (12.42 to 22.08) |
|  | AT | 49.97  (45.54 to 54.40) | 39.67  (35.06 to 44.28) | 40.15  (35.62 to 44.68) | 30.93  (26.06 to 35.80) |
|  | Difference | -2.77  (-9.01 to 3.47) | -8.37  (-14.86 to -1.88) | -16.47  (-22.84 to -10.11) | -13.68  (-20.54 to -6.82) |
|  | *P*-value | 0.381 | 0.012* | <0.001*** | <0.001*** |
| PGIC | MSAT | - | - | 2.02  (1.81 to 2.22) | 2.20  (1.96 to 2.43) |
|  | AT | - | - | 2.80  (2.60 to 3.01) | 2.65  (2.41 to 2.89) |
|  | Difference | - | - | -0.79  (-1.08 to -0.49) | -0.45  (-0.79 to -0.12) |
|  | *P*-value | - | - | <0.001*** | 0.009** |

***MSAT***, Motion Style Acupuncture Treatment; **AT**, Acupuncture Treatment; ***VAS***, Visual Analog Scale; ***NRS***, Numeric Rating Scale; ***EQ-5D***-***5L,*** EuroQol 5-Dimension 5-level; ***SF-12,*** 12-item Short-Form Health Survey; ***PCS,*** Physical Component Summary; ***MCS***, Mental Component Summary; ***PGIC***, Patient Global Impression of Change; ***EQ-VAS***, EuroQol-5 dimension visual analog scale; ***NDI***: Neck Disability Index; ***NPQ***: Northwick Park Neck Pain Questionnaire; ***WPAI***; Work Productivity and Activity Impairment Questionnaire

| Supplementary Table 8. Area under the Curve of outcome according to Treatment | | | |  |
| --- | --- | --- | --- | --- |
|  | MSAT | Control | Difference (95% CI) | P value |
| **ITT set** |  |  |  |  |
| VAS on movement | 203.47  (175.65 to 231.29) | 312.32  (284.42 to 340.22) | -108.85  (-148.33 to -69.38) | <0.001*** |
| VAS at rest | 162.03  (135.94 to 188.13) | 250.27  (224.03 to 276.51) | -88.24  (-125.30 to -51.17) | <0.001*** |
| NRS on movement | 20.36  (17.64 to 23.08) | 30.33  (27.60 to 33.06) | -9.97  (-13.82 to -6.12) | <0.001*** |
| NRS at rest | 15.70  (13.11 to 18.28) | 24.81  (22.21 to 27.41) | -9.11  (-12.78 to -5.44) | <0.001*** |
| NPQ | 167.67  (149.94 to 185.40) | 215.20  (197.49 to 232.91) | -47.53  (-72.68 to -22.39) | <0.001*** |
| NDI | 124.14  (108.24 to 140.03) | 168.16  (152.28 to 184.05) | -44.02  (-66.47 to -21.58) | <0.001*** |
| EQ-5D-5L | 6.68  (6.56 to 6.80) | 6.49  (6.37 to 6.61) | 0.19  (0.02 to 0.36) | 0.032* |
| EQ-VAS | 592.89  (570.22 to 615.57) | 560.32  (537.61 to 583.02) | 32.57  (0.57 to 64.58) | 0.046* |
| PCS | 389.99  (380.81 to 399.17) | 375.77  (366.56 to 384.98) | 14.22  (1.24 to 27.20) | 0.032* |
| MCS | 403.00  (391.87 to 414.13) | 399.49  (388.37 to 410.61) | 3.51  (-12.35 to 19.38) | 0.662 |

***MSAT***, Motion Style Acupuncture Treatment; ***VAS***, Visual Analog Scale; ***NRS***, Numeric Rating Scale; ***EQ-5D***-***5L,*** EuroQol 5-Dimension 5-level; ***PCS,*** Physical Component Summary; ***MCS***, Mental Component Summary; ***EQ-VAS***, EuroQol-5 dimension visual analog scale; ***NDI***: Neck Disability Index; ***NPQ***: Northwick Park Neck Pain Questionnaire; ***ITT***, Intention-to-treat

Supplementary Figure 1 Subgroup Analysis of the Primary Outcome According to Baseline Characteristics


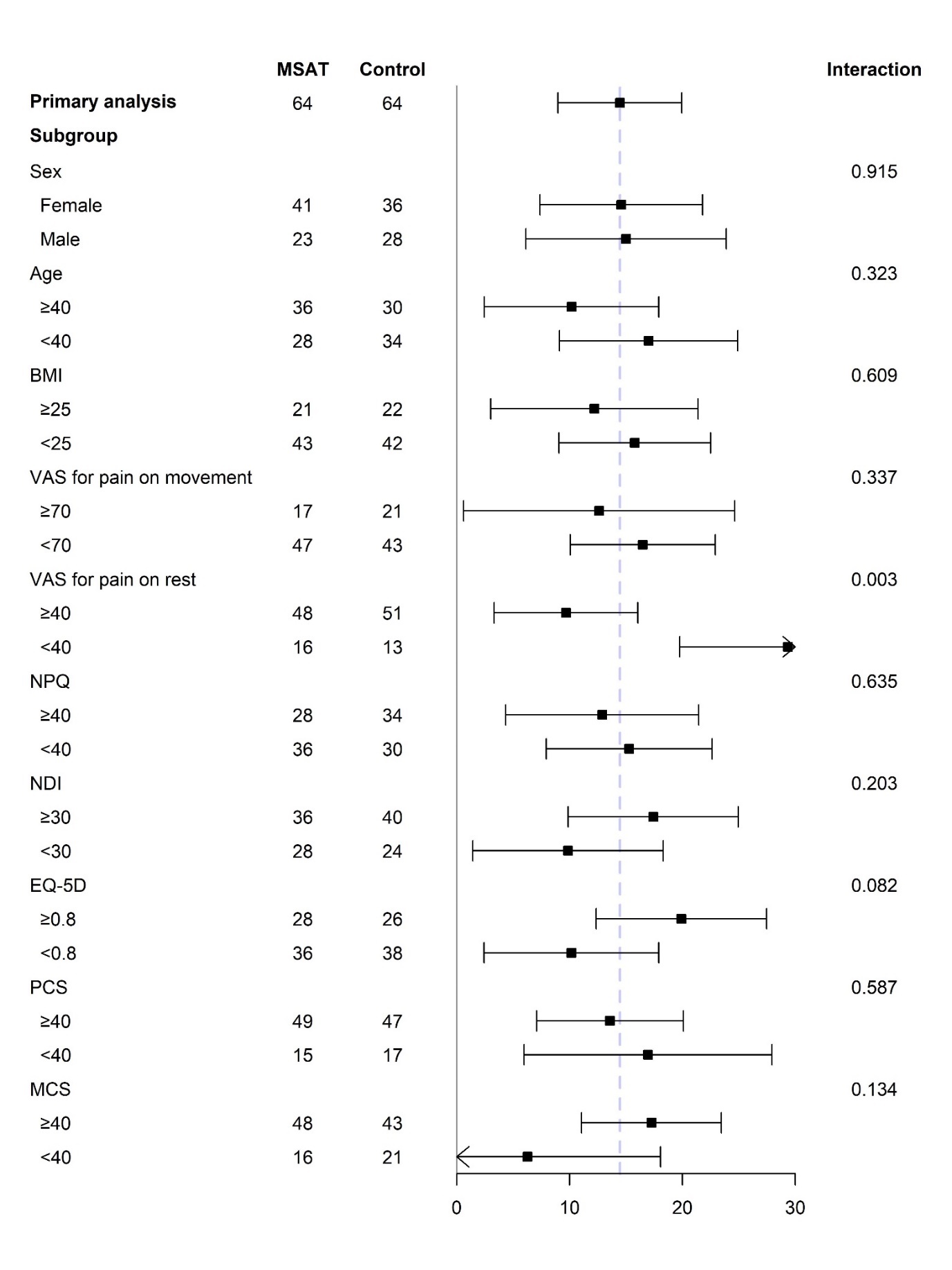
Forest plot showing subgroup analysis of the primary outcome (change in VAS for neck pain during movement). Values to the right of the vertical line indicate greater improvement with MSAT compared to control, while values to the left indicate greater improvement with control treatment

***MSAT***, Motion Style Acupuncture Treatment; ***BMI****,* Body mass index; ***VAS***, Visual Analog Scale; ***EQ-5D***-***5L,*** EuroQol 5-Dimension 5-level; ***PCS,*** Physical Component Summary; ***MCS***, Mental Component Summary; ***EQ-VAS***, EuroQol-5 dimension visual analog scale; ***NDI***: Neck Disability Index; ***NPQ***: Northwick Park Neck Pain Questionnaire;

.
